# Targeted whole-viral genome sequencing from formalin-fixed paraffin-embedded neuropathology specimens

**DOI:** 10.1007/s00401-024-02812-z

**Published:** 2024-10-09

**Authors:** Charlotte Gorißen, Anne Albers, Viktoria Ruf, Emil Chteinberg, Reiner Siebert, Leonille Schweizer, Lukas Kaufmann, Joachim E. Kühn, Dennis Tappe, Tanja Kuhlmann, Christian Thomas

**Affiliations:** 1 Institute of Neuropathology, University Hospital Münster, Münster, Germany; 2 Center for Neuropathology and Prion Research, Faculty of Medicine, LMU Munich, Munich, Germany; 3 Institute of Human Genetics, Ulm University and Ulm University Medical Center, Ulm, Germany; 4 Edinger Institute (Institute of Neurology), University Hospital Frankfurt, Goethe University, Frankfurt Am Main, Germany; 5 Institute of Virology-Clinical Virology, University Hospital Münster, Münster, Germany; 6 Bernhard Nocht Institute for Tropical Medicine, Hamburg, Germany

**Keywords:** Virus, Sequencing, Formalin-fixed paraffin-embedded

Viral infections of the central nervous system (CNS) can be severe and potentially life-threatening diseases [4]. Precise detection of the causative pathogen is crucial for definite diagnosis, prognosis and related therapy decisions. In routine clinical practice, serologic testing and viral PCR remain the gold standard, although a causative agent is not identified in more than 50% of patients with acute meningoencephalitis [4, 5]. Conventional metagenomic next-generation sequencing represents a hypothesis-free alternative, but is often hampered by a high host background and low viral coverage [1]. Metagenomics has been evaluated in cerebrospinal fluid [10], but depending on the spatial dynamics of CNS infections, viral nucleic acids might only be detectable in brain tissue specimens [11]. Native or fresh-frozen tissue is not available in most cases, and archival formalin-fixed paraffin-embedded (FFPE) samples typically contain highly degraded, low-quality nucleic acids. Hybridization-capture next-generation sequencing (NGS) has been successfully applied to enrich distinct regions of the human genome [2], but can also be used to target pathogen-specific sequences [3]. We thus conducted a proof-of-concept study to assess a viral nucleic acid enrichment protocol for FFPE specimens.

After nucleic acid extraction from 23 FFPE samples (plus 5 non-FFPE controls) with confirmed viral infection, target enrichment was performed using the Comprehensive Viral Research Panel (Twist Bioscience). All samples were sequenced (i) with and (ii) without viral enrichment (i.e., conventional metagenomics) on a NextSeq device, respectively. Bioinformatic analysis was performed using a custom pipeline (https://github.com/ctho1/metagenomics_pipeline). A detailed description of our protocol is available in Supplementary Methods.

The median age of the 7 females and 16 males was 64 years (range, 7–80 years). Samples comprised CNS infectious diseases caused by DNA (*n* = 10) and RNA viruses (*n* = 13), including herpes simplex virus 1 (HSV-1, *n* = 5), JC polyomavirus (JCPyV, *n* = 4), tick-borne encephalitis virus (TBEV, *n* = 3), Borna disease virus 1 (BoDV-1, *n* = 9), human immunodeficiency virus 1 (HIV-1, *n* = 1) and Epstein–Barr virus (EBV, *n* = 1). To assess the potential for detecting viral sequences, we tested our protocol with five non-FFPE samples (2 EBV-positive Burkitt lymphoma cell lines and 3 SARS-CoV-2-positive nasopharyngeal swabs, Supplementary Table 1). Compared to conventional metagenomics (i.e. random or ‘shot-gun’ sequencing), application of the viral target enrichment led to a median 200-fold enrichment of viral sequences per million reads (Supplementary Fig. 1). Notably, alignment to the corresponding viral reference genomes achieved a median genome coverage of 99.75%, and de novo assembly (median N50: 29819 bp) facilitated complete genome reconstruction in all SARS-CoV-2 cases with a single contig (Supplementary Table 1). High coverage and paired-end sequencing mode also allowed for detection of viral integration sites into the human genome (Supplementary Methods) including the well-known insertions of the EBV genome into Chr1 of the Burkitt lymphoma cell line Namalwa [8] (Supplementary Table 2) that was missed without viral enrichment. We next applied the protocol to all FFPE specimens (7 biopsy and 16 autopsy cases) with an average FFPE storage duration of 6 years (range, 1–22 years; Supplementary Table 1). Sequencing runs produced an average of 26.3 million reads for conventional metagenomics and 24.4 million reads for the viral enrichment panel (Fig. 1). As expected, conventional metagenome sequencing was associated with a high host background (average non-viral reads: 99%). The viral enrichment panel includes probes targeting > 3000 viral genomes, including EBV, HSV-1, JCPyV, TBEV, HIV-1 and HSV-1 (samples #1–14). Taxonomic classification of these samples demonstrated a considerably lower background compared to conventional metagenomics (mean: 53% vs. 99%, *p* < 0.001, *t* test) with a higher proportion of viral reads per million (median: 241509 vs. 344 rpm, *p* = 0.001, *t* test, Supplementary Table 1, Fig. 1b) resulting in a median 1129-fold enrichment of viral sequences. While conventional metagenomics achieved a median genome coverage of 72% and a median depth of 3X, viral enrichment improved these results significantly, enabling a median genome coverage of 99% and a median depth of 8174X (Fig. 1, Supplementary Table 1). Consequently, the viral panel provided > 85% genome coverage in 10/12 samples (Fig. 1c). Moreover, de novo assembly resulted in larger contiguous fragments compared to conventional metagenomics (mean N50: 12257 vs. 1741 bp, *p* = 0.03, *t* test). BoDV-1 (samples #15–23) are not specifically targeted by the panel, resulting in lower median viral genome coverage compared to conventional metagenomics (58% vs. 97%, Fig. 1a). Viral integration events into the host genome were predicted in 9/23 samples (Supplementary Table 2) with 3/5 JCPyV samples showing high confidence integration events (with human-viral chimeric reads and discordant paired-end reads) in multiple chromosomes. In addition to the known viruses, a manageable number of other viruses were also detected with both methods (Supplementary Table 3), some of which were confirmed by PCR (EBV in sample #4, HPV16 in sample #1 and JCPyV in sample #11, Supplementary Table 4). However, manual inspection of viral alignments suggested index hopping in sample #7 (Supplementary Fig. 2), and JCPyV could not be confirmed by PCR in this sample, highlighting the need for confirmatory testing when unexpected results are encountered.

**Fig. 1:**
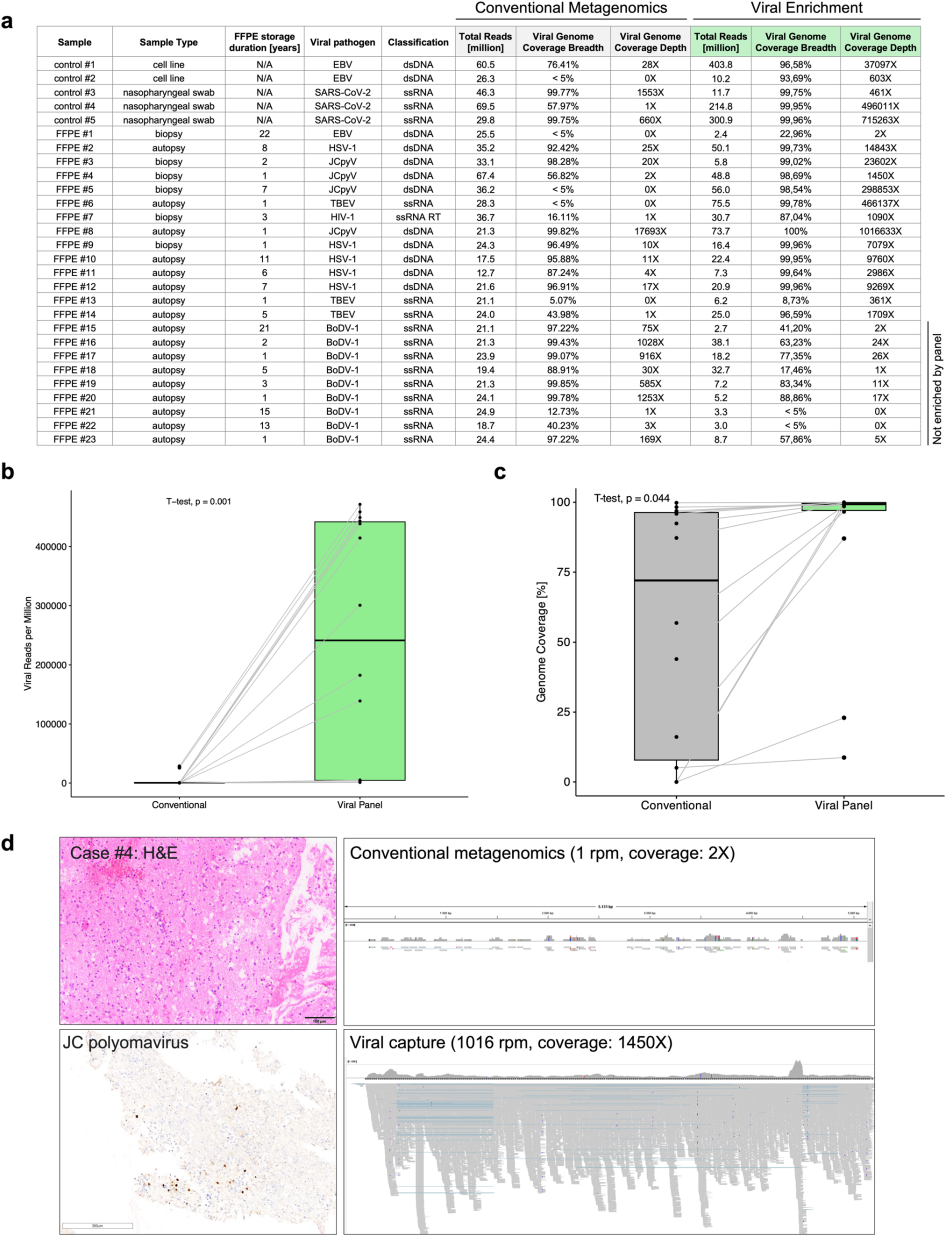
Sample overview and results of viral metagenomic sequencing analysis. **a** Overview of the cohort with sample and virus characteristics as well as analysis results of direct (conventional) metagenomic sequencing and viral panel sequencing. **b** In FFPE samples #1–#14 (which contained viruses targeted by the panel), viral capture sequencing demonstrated a significantly higher proportion of viral reads per million (median: 241509 vs. 344 rpm), leading to a median 1129-fold enrichment of viral sequences compared to conventional metagenomics (*p* = 0.001, paired *t* test). **c** Consistent with the increased proportion of viral reads, viral genome coverages are significantly higher in samples #1 to #14 when using the viral capture panel compared to conventional metagenomics (*p* = 0.044, paired *t* test). **d** Illustration of case #4 with progressive multifocal leukoencephalopathy (PML) due to JC virus (JCPyV) infection. Immunohistochemistry demonstrates only a few scattered JC virus-positive cells. Conventional metagenomics resulted in 1 JCPyV read per million and an incomplete genome coverage, whereas viral enrichment sequencing led to a 1000-fold enrichment: 1016 JCPyV reads per million and an average genome coverage depth of 1450X. *HSV-1* herpes simplex virus 1, *JCPyV* JC polyomavirus, *TBEV* tick-borne encephalitis virus, *BoDV-1* Borna disease virus 1, *HIV-1* human immunodeficiency virus 1, *EBV* Epstein–Barr virus

Taken together, metagenomic sequencing of virus-enriched libraries from FFPE specimens represents a valuable method for viral whole-genome sequencing. The strong enrichment (median > 1000-fold) allowed a highly sensitive detection of viral pathogens even in low-biomass samples with high host background [1]. Near-complete viral genomes allow for more precise and refined strain-level classification that can be used to trace outbreaks and host integration events, but also detection of intra-host evolution and clinically important antiviral-resistant variants [6]. Moreover, the approach applies well to archival FFPE tissues (oldest sample in our study: 22 years) and thus enables sequencing of retrospective cohorts to address scientific questions such as the role of viral pathogens in neurological disease or in conditions such as sudden infant death syndrome [9]. The method enables the detection of unexpected viruses (like JCPyV in sample #11), but manual inspection and confirmatory testing are necessary, especially when similar viruses are found in the same run. These findings may arise from index hopping, a phenomenon that standard analysis pipelines cannot detect. Thus, using a negative control or a synthetic control sample with known viruses and further confirmation of unexpected results with orthogonal methods are recommended, especially in clinical settings. In our cohort, 3/5 cases with JC polyomavirus infection showed high evidence of chromosomal integration, a phenomenon well known from the closely related BK polyomavirus [7] but thus far unrecognized in the human brain. While the panel targets a broad range of viral species, bornaviruses are not specifically enriched. The detection of BoDV-1 reads with significant coverage in seven out of nine samples is likely due to carryover from the original library. Due to the similarity of the library preparation process, viral enrichment is easily applicable to laboratories that already perform hybrid-capture sequencing. In conclusion, metagenome sequencing with a viral capture panel represents a valuable method to perform whole-viral genome sequencing across a broad variety of viral species from low-quality and low-biomass FFPE material.

## Supplementary Information

Below is the link to the electronic supplementary material.

Supplementary file1 (DOCX 29 KB)

Supplementary file2 (PPTX 427 KB)

Supplementary file3 (XLSX 100 KB)
