# Supplementary material for: Targeted whole-viral genome sequencing from formalin-fixed paraffin-embedded neuropathology specimens
Source: Acta Neuropathol. 2024 Oct 9;148(1):51. doi: 10.1007/s00401-024-02812-z (PMC11464609; doi:10.1007/s00401-024-02812-z)
Supplement: Supplementary file 1 — Supplementary file1 (DOCX 29 KB) [file 401_2024_2812_MOESM1_ESM.docx]

**Supplementary methods**

***Materials:*** Formalin-fixed paraffin-embedded (FFPE) samples of 23 specimens with confirmed viral encephalitis were retrieved from the Institutes of Neuropathology Münster, Munich and Frankfurt. Quantification of viral load is mentioned in Supplementary Table 1 where applicable. In addition, DNA from two EBV-positive Burkitt lymphoma cell lines (Daudi and Namalwa) from the Institute of Human Genetics in Ulm and TNA (total nucleic acids) from three nasopharyngeal swabs positive for SARS-CoV-2 from the Institute of Virology in Münster were investigated. The use of biopsy specimens for research upon anonymization was in accordance with local regulations of the University Hospitals Münster, Frankfurt and Munich and approved by the Münster ethics committee (2023-491-f-S).

***DNA Isolation:*** Genomic DNA was isolated from FFPE material using the Maxwell RSC FFPE Plus DNA Kit (Promega, Madison, USA). All DNA samples were eluted in 50 µl 1x TE Buffer pH 7.5 (Promega, Madison, USA). Genomic DNA was quantified with the QuantiFluor ONE dsDNA System (Promega, Madison, USA). DNA integrity (DIN) values and mass concentration of gDNA were determined using the Genomic DNA ScreenTape Assay for the TapeStation (Agilent Technologies, Santa Clara, USA) with the adjusted region setting of 50 bp to >60,000 bp.

***RNA Isolation:*** RNA extraction from FFPE tissue was carried out using the Maxwell CSC RNA FFPE Kit (Promega). RNA concentration and DV200 data were measured using an RNA ScreenTape Assay for the TapeStation (Agilent Technologies, Santa Clara, USA).

***cDNA synthesis:*** Before library preparation, RNA was transcribed into cDNA according to the Total Nucleic Acids Library Preparation Kit for Viral Panel Pathogen Detection and Characterization protocol (Twist Bioscience, San Francisco, USA) with the ProtoScript II First Strand cDNA Synthesis Kit, NEBNext Ultra II Non-Directional RNA Second Strand Synthesis Module and Random Primer 6 (all New England Biolabs, Ipswich, USA). DIN values and concentration of cDNA were determined using the Genomic DNA ScreenTape Assay for the TapeStation (Agilent Technologies, Santa Clara, USA) with the adjusted region setting of 50 bp to >60,000 bp.

***Conventional Metagenomics:*** For library preparation, enzymatic fragmentation of cDNA or gDNA, respectively, was performed with adjusted incubation times depending on the DIN value (DIN ≥5.1: 20min; DIN 4.0-5.0: 15min; 3.5-3.9: 10min; DIN 2.0-3.4: 7.5min; DIN <2: 5min). After universal adapter ligation, random PCR amplification using Twist UDI primers was performed. The number of PCR cycles for each sample was determined by the DIN values (DIN ≥5.1: 8 cycles; DIN 2.0-5.0: 11 cycles; DIN <2: 12 cycles). Quality control and estimation of concentration of individual sample libraries were performed with the D1000 ScreenTape Assay for the TapeStation (Agilent Technologies, Santa Clara, USA) and sequencing was performed on a NextSeq device (Illumina, San Diego, USA) in paired-end mode (2 x 75bp).

***Target enrichment with viral panel:*** Amplified patient libraries from conventional metagenomics were pooled per 3 – 16 samples. Hybridization of pooled libraries was performed with the Twist Comprehensive Virus probe panel (Twist Bioscience, San Francisco, USA), consisting of ∼1 million 120 bp probes targeting 15,488 different viral strains infecting human and animals [7]. Hybridization was performed for 16 hours of incubation followed by several wash steps. Captured fragments were further amplified by a post-hybridization PCR (12 cycles). Finally, captured libraries were purified by a bead clean up using AmpureXP, and quantity and fragment size were determined with the D1000 ScreenTape Assay for the TapeStation (Agilent Technologies, Santa Clara, USA) and sequencing was performed on a NextSeq device (Illumina, San Diego, USA) in paired-end mode (2 x 75bp).

***Data analysis:*** Data generated from conventional metagenomics and viral panel sequencing was processed with the same bioinformatics pipeline. Trimming and filtering of low-quality and low-complexity reads was performed with fqtrim. The remaining high-quality reads were aligned to the human reference genome (hg38) using Bowtie 2 [3]. Unaligned (non-human) reads were next subjected to several metagenomic classification tools. First, taxonomic classification with Centrifuge [2] and Kraken 2 [6] using different reference databases (centrifuge: nt [all NCBI nucleotide non-redundant sequences], Kraken 2: k2_standard_20230314 and k2_viral_20230314) was performed. Next, reads were aligned against ~12,000 RefSeq virus genomes (as well as ~4,500 human-infecting virus strains related to the RefSeq viruses) using STAR [1] and the STAR_index_GRCh37viral_GENCODE19 reference (https://github.com/suhrig/arriba). Phages, endogenous retroviruses and plant viruses were discarded. Detection of viral integration sites into the human genome was performed using Arriba [5] with the same STAR_index_GRCh37viral_GENCODE19 index. The tool considers both split reads, which directly span the junctions between human and viral sequences, and discordant reads, where paired-end reads align to different genomes or unexpected positions. True predictions usually have a balanced number of split reads and discordant mates. Events with only discordant mates or without discordant mates and only split reads having anchors in just one gene are frequently artifacts. The columns “split_read1” and “split_read2” in Supplementary Table 2 indicate the number of supporting split reads anchored in gene1 and gene2, respectively (the anchor is defined as the gene where the longer segment of the split read aligns). Reads with ≥ 1 split_read1 and ≥ 1 split_read2 as well as ≥ 1 discordant read are considered high-confidence integration candidates. *De novo* assembly of viral reads into larger contigs was performed using MEGAHIT [4]. Contigs > 1000 bp were classified with Kraken 2 and Centrifuge using the aforementioned references. Contigs were manually analyzed with NCBI BLAST (https://blast.ncbi.nlm.nih.gov/Blast.cgi). Custom scripts for the bioinformatics pipeline are available on github (https://github.com/ctho1/metagenomics_pipeline).

***Data availability:*** Host-filtered fasta files are available under the BioProject Accession No. PRJNA1134792 (https://www.ncbi.nlm.nih.gov/bioproject/PRJNA1134792/).

**References**

1. Dobin A, Davis CA, Schlesinger F, Drenkow J, Zaleski C, Jha S, Batut P, Chaisson M, Gingeras TR (2013) STAR: ultrafast universal RNA-seq aligner. Bioinformatics 29:15–21. doi: 10.1093/bioinformatics/bts635

2. Kim D, Song L, Breitwieser FP, Salzberg SL (2016) Centrifuge: rapid and sensitive classification of metagenomic sequences. Genome Res 26:1721–1729. doi: 10.1101/gr.210641.116

3. Langmead B, Salzberg SL (2012) Fast gapped-read alignment with Bowtie 2. Nat Methods 9:357–359. doi: 10.1038/nmeth.1923

4. Li D, Liu C-M, Luo R, Sadakane K, Lam T-W (2015) MEGAHIT: an ultra-fast single-node solution for large and complex metagenomics assembly via succinct de Bruijn graph. Bioinformatics 31:1674–1676. doi: 10.1093/bioinformatics/btv033

5. Uhrig S, Ellermann J, Walther T, Burkhardt P, Fröhlich M, Hutter B, Toprak UH, Neumann O, Stenzinger A, Scholl C, Fröhling S, Brors B (2021) Accurate and efficient detection of gene fusions from RNA sequencing data. Genome Res 31:448–460. doi: 10.1101/gr.257246.119

6. Wood DE, Lu J, Langmead B (2019) Improved metagenomic analysis with Kraken 2. Genome Biol 20:257. doi: 10.1186/s13059-019-1891-0

7. Twist Comprehensive Viral Research Panel Strain and Species List. https://www.twistbioscience.com/resources/technical-document/twist-comprehensive-viral-research-panel-strain-and-species-list. Sep 2024
