# Supplementary material for: Targeted whole-viral genome sequencing from formalin-fixed paraffin-embedded neuropathology specimens
Source: Acta Neuropathol. 2024 Oct 9;148(1):51. doi: 10.1007/s00401-024-02812-z (PMC11464609; doi:10.1007/s00401-024-02812-z)
Supplement: Supplementary file 2 — Supplementary file2 (PPTX 427 KB) [file 401_2024_2812_MOESM2_ESM.pptx]

## Slide 1
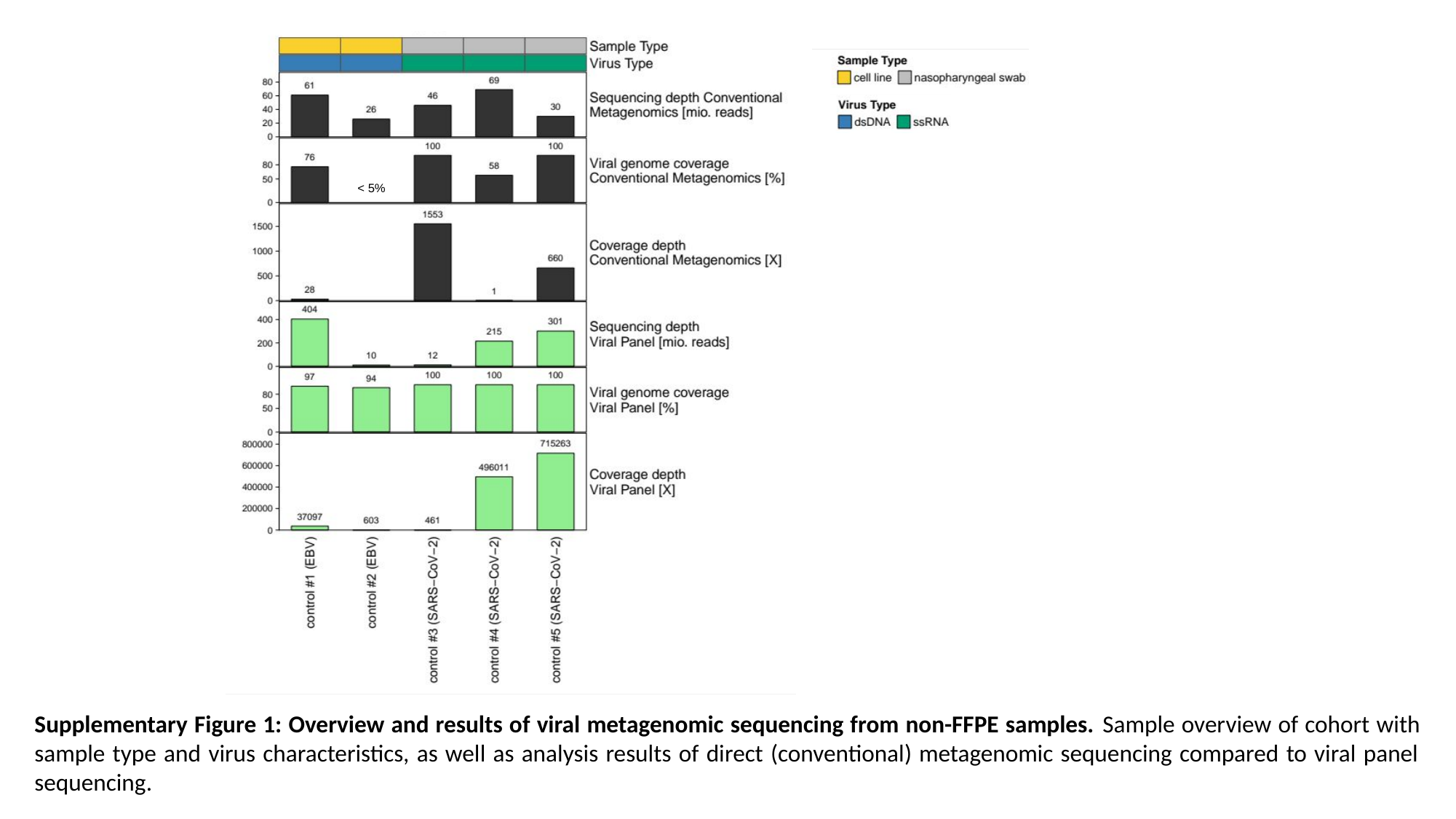

< 5%
Supplementary Figure 1: Overview and results of viral metagenomic sequencing from non-FFPE samples. Sample overview of cohort with sample type and virus characteristics, as well as analysis results of direct (conventional) metagenomic sequencing compared to viral panel sequencing.

## Slide 2
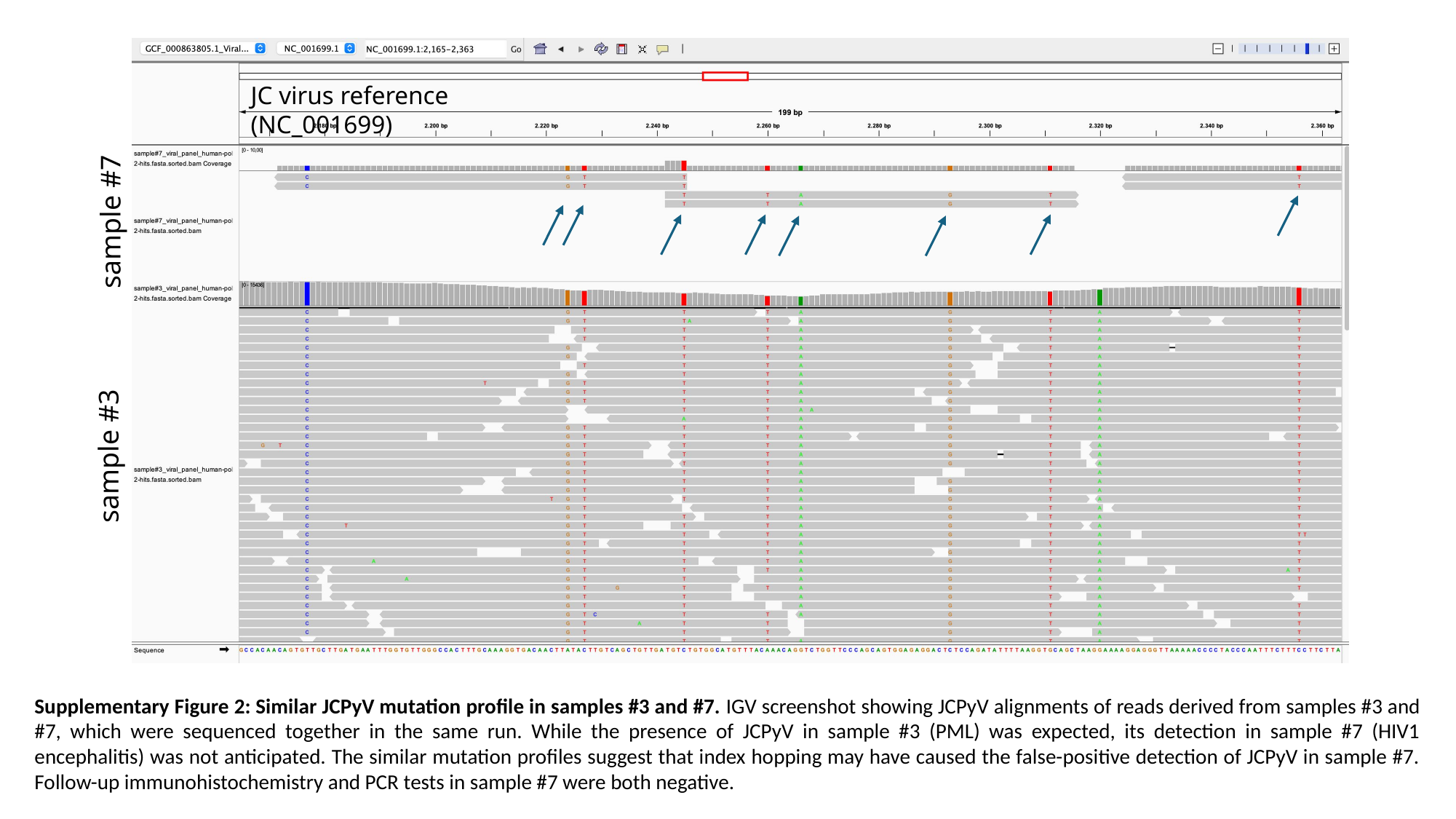

JC virus reference (NC_001699)
sample #7
sample #3
Supplementary Figure 2: Similar JCPyV mutation profile in samples #3 and #7. IGV screenshot showing JCPyV alignments of reads derived from samples #3 and #7, which were sequenced together in the same run. While the presence of JCPyV in sample #3 (PML) was expected, its detection in sample #7 (HIV1 encephalitis) was not anticipated. The similar mutation profiles suggest that index hopping may have caused the false-positive detection of JCPyV in sample #7. Follow-up immunohistochemistry and PCR tests in sample #7 were both negative.
